# Local radiotherapy for murine breast cancer increases risk of metastasis by promoting the recruitment of M-MDSCs in lung

**DOI:** 10.1186/s12935-023-02934-6

**Published:** 2023-06-02

**Authors:** Zhengzheng Zhang, Zhiyan Yao, Zimeng Zhang, Ling Cui, Ling Zhang, Gang Qiu, Xiaotian Song, Shuxia Song

**Affiliations:** 1 Department of Immunology, Hebei Medical University, Shijiazhuang, China; 2 Hebei province Key Laboratory of Immunological mechanism and intervention of serious diseases, Hebei Medical University, Shijiazhuang, China; 3 Department of Oncology, Hebei People’s Hospital, Shijiazhuang, China

**Keywords:** Radiotherapy, Premetastatic niche, M-MDSC, GM-CSF, CCL2/CCR2

## Abstract

**Background:**

Radiotherapy is one of the effective methods for treatment of breast cancer; however, controversies still exist with respect to radiotherapy for patients with TNBC. Here, we intend to explore the mechanism by which local radiotherapy promotes the recruitment of M-MDSCs in the lung and increases the risk of lung metastasis in TNBC tumor-bearing mice.

**Methods:**

A single dose of 20 Gy X-ray was used to locally irradiate the primary tumor of 4T1 tumor-bearing mice. Tumor growth, the number of pulmonary metastatic nodules, and the frequency of MDSCs were monitored in the mice. Antibody microarray and ELISA methods were used to analyze the cytokines in exosomes released by irradiated (IR) or non-IR 4T1 cells. The effects of the exosomes on recruitment of MDSCs and colonization of 4T1 cells in the lung of normal BALB/c mice were observed with the methods of FCM and pathological section staining. T lymphocytes or 4T1 cells co-cultured with MDSCs were performed to demonstrate the inhibitory effect on T lymphocytes or accelerative migration effect on 4T1 cells. Finally, a series of in vitro experiments demonstrated how the exosomes promote the recruitment of M-MDSCs in lung of mice.

**Results:**

Even though radiotherapy reduced the burden of primary tumors and larger lung metastatic nodules (≥ 0.4 mm^2^), the number of smaller metastases (< 0.4 mm^2^) significantly increased. Consistently, radiotherapy markedly potentiated M-MDSCs and decreased PMN-MDSCs recruitment to lung of tumor-bearing mice. Moreover, the frequency of M-MDSCs of lung was positively correlated with the number of lung metastatic nodules. Further, M-MDSCs markedly inhibited T cell function, while there was no difference between M-MDSCs and PMN-MDSCs in promoting 4T1 cell migration. X-ray irradiation promoted the release of G-CSF, GM-CSF and CXCl1-rich exosomes, and facilitated the migration of M-MDSCs and PMN-MDSCs into the lung through CXCL1/CXCR2 signaling. While irradiated mouse lung extracts or ir/4T1-exo treated macrophage culture medium showed obvious selective chemotaxis to M-MDSCs. Mechanistically, ir/4T1-exo induce macrophage to produce GM-CSF, which further promoted CCL2 release in an autocrine manner to recruit M-MDSCs via CCL2/CCR2 axis.

**Conclusions:**

Our work has identified an undesired effect of radiotherapy that may promote immunosuppressive premetastatic niches formation by recruiting M-MDSCs to lung. Further studies on radiotherapy combined CXCR2 or CCR2 signals inhibitors were necessary.

**Supplementary Information:**

The online version contains supplementary material available at 10.1186/s12935-023-02934-6.

## Introduction

As a conventional treatment for breast cancer and other tumors, radiotherapy (RT) is widely used in clinical practice, and many patients benefit from it [1, 2]. But there are some problems with radiation that can’t be ignored, for example, it has been reported that current RT techniques not only hard to clear CTCs effectively, but also may increase the number of CTCs by inducing tumor cells to develop EMT [3–6]. RT can also cause inflammatory reactions in patients (hosts), especially in the lungs [7, 8], which has been shown to be associated with a variety of cytokines, such as interleukins (IL)-4, IL-6, IL-10, IL-13, IL-17, and IL-18 [9]. RT also causes other side effects such as leukopenia [10]. These drawbacks of RT may become the hidden danger of tumor recurrence or spread. Nearly 15% of patients still has recurrence or metastasis within 5 years after RT [10, 11], especially in patients with stage T3 disease, RT showed no benefit in terms of improved survival [12]. Therefore, the benefits of RT do not preclude the risk of recurrence or metastasis from the negative effects of radiation on the cancer cell or host.

CTCs have been found in the peripheral blood of patients with a variety of tumors, and RT, chemotherapy and even needle biopsy may promote the formation of CTCs. Although the number of CTCs is independent of the size of the primary tumor and only a fraction of CTCs is capable of surviving, CTCs may potential to generate productive local or distant metastases with the mechanism of “tumor self-seeding” [13]. It has been reported that RT can enhance the number of CTCs and significantly facilitate the invasiveness of non-small cell lung cancer [14, 15]. Although CTCs as a potential predictive biomarker for benefit of adjuvant radiotherapy in early breast cancer was recently reported [16], ENPP1^hi^ circulating tumor cells (CTCs) contribute to relapse by a self-seeding mechanism [13]. Inhibition of radiation-induced EMT by α lipoic acid can remarkably reduce the risk of recurrence or metastasis by reducing CTCs formation [17]. In addition, reducing lung damage or inflammation post-radiation also reduces the risk of lung metastasis of breast cancer [8, 11]. Therefore, radiotherapy, as a conventional tumor treatment at present, may be a double-edged sword for the treatment of tumors including breast cancer, which may lead to the risk of tumor recurrence or metastasis due to increased CTCs or inflammation [18, 19].

Tumor-derived exosomes support formation of pre-metastatic niche, which is conducive to the implantation of CTCs in remote organs [20, 21]. Recent studies have revealed that breast cancer cells secrete exosomes contributed to the generation of pre-metastatic niche to promote bone metastasis [21]. Bone marrow-derived cells (BMDCs) are the main cellular components of pre-metastatic niche, including MDSCs [22]. MDSCs are a chief component of tumor microenvironment, local inflammation may be an important factor for recruitment of MDSCs into pre-metastasis niche [23]. Generally, tumor-derived exosomes can promote the differentiation and inhibitory function of MDSCs by carrying a variety of factors [23–26]. Therefore, the number of MDSCs cells can reflect the immune imbalance and tumor development in the microenvironment. Several recent studies have shown that MDSCs can be classified into two subpopulations by the phenotype and morphology, namely M-MDSCs with the phenotype CD11b^+^Gr-1^+^F4/80^+^ (or CD11b^+^Ly6G^−^Ly6C^hi/+^) and PMN-MDSCs with the phenotype CD11b^+^Gr-1^+^F4/80^−^ (or CD11b^+^Ly6G^+^Ly6C^int/+^) [27]. Both subtypes of MDSCs have plasticity, and MDSCs differentiation is highly dependent on specific microenvironment in response to various growth factors and cytokines associated with cancer or stromal cells [28]. In particular, the CCL2/CCR2 or CXCR2/CXCL1 pathway has been demonstrated to play a critical role in the migration of M-MDSCs or PMN-MDSCs to tumors [29, 30]. In this study, the 4T1 tumor-bearing mouse model prone to lung metastasis is used to investigate the effects of local RT on MDSCs recruitment to lung with the aim to explore the possible mechanism of RT-induced MDSCs recruitment to form premetastatic niche. We found that local RT can increase the number of small metastatic nodules and the infiltration of M-MDSCs in lung. In addition, X-ray irradiation could markedly potentiate 4T1 cells to produce GM-CSF/G-CSF/CXCL1-rich exosomes. On one hand, M-MDSCs were able to be mobilized to migrate into lung to form pre-metastatic niches by CXCR2/CXCL1 signal provided in part by exosomes. On the other hand, the exosomes may induce macrophages to produce GM-CSF, which induces CCL2 release in an endocrine manner and further promotes M-MDSCs recruitment by CCR2/CCL2 pathway. These findings reveal an undesired effect of local RT that may contribute to form immunosuppressive pre-metastatic niche, which might hinder the efficacy of RT treatment and contribute to danger of lung metastasis.

## Materials and methods

### Cell culture

4T1 cell line (ATCC, CRL-2539) was purchased from National Collection of Authenticated Cell Cultures (Shanghai, China). The cell line was maintained in RPMI 1640 (Thermo Scientific-CN), supplemented with 10% FBS (HyClone, Beijing wobisen technology co., LTD, China) and antibiotic/antimycotic 10,000 U/ml.

### Mouse model

Female BALB/c mice (8-10-weeks old) were purchased from the Ex&InVivo Biotechnology company (Shijiazhuang, China). All mice were housed in a specific pathogen-free environment under protocols approved by the Animal Care Committee of Hebei Medical University, China, and all experiments related to the mice were performed in accordance with the approved guidelines.

All animal experiments were done according to a protocol approved by the Institutional Animal Care and Use Committee. 1 × 10^6^ 4T1 cells were inoculated into the fourth mammary fat pad on the right of the mice (50 µl/mouse). 10 days after tumor inoculation, when the tumor diameter was about 7 mm, local RT was performed with a single dose of 0 or 20 Gy. Radiotherapy was performed by using a 225 kVp cabinet X-ray system. Animals were anesthetized and placed under a 3.2-mm lead shield with a 1-centimeter round hole to focus the tumor.

### Isolation of M-MDSCs and PMN-MDSCs

MDSCs were isolated from single cell suspensions of the right lobe lung tissues of 4T1 tumor-bearing mice. Firstly, single cells of lung tissue were isolated by mechanical method, and then Ficoll gradient centrifugation method was used to obtain the mononuclear cells. Then, the single mononuclear cells were labeled with anti-mouse CD16/CD32 antibody for 30 min, and subsequently followed staining by 488 conjugated anti-CD11b murine antibodies for another 30 min. After incubation, CD11b positive cells were isolated with a FACS Aria cell sorter. The CD11b^+^ cells were divided into two tubes and then the PE conjugated anti-Ly6C or Ly6G antibody was added for 30 min. Finally, the anti-PE microbeads were added and M-MDSCs or PMN-MDSCs were isolated per the manufacturer’s protocol. The purity of the M-MDSCs and PMN-MDSCs was assessed by flow cytometry and found to be ≥ 99.8%.

### Flow cytometry

To analyze the frequency of M-MDSCs, PMN-MDSCs or macrophages in lung of 4T1 tumor-bearing mice, the single mononuclear cells from lung tissue were labeled with CD32/16 antibodies for 30 min, and followed with 488-conjugated CD11b (dilution 1/400), APC-conjugated Gr-1 (dilution 1/100) and PE-conjugated F4/80 (dilution 1/200) antibodies for another 30 min. The flow cytometry data were acquired using BD LSRII flow cytometer and analyzed by FlowJo Software (Tree Star).

To analyze the CXCR2 or CCR2 expression in MDSCs, the single mononuclear cells from lung tissue were labeled with APC-conjugated CXCR2 or CCR2 antibody following CD32/16 antibodies blocking.

### Cytokine array and ELISA

The presence of cytokines in the exosomes released by 4T1 cells were detected using the Mouse Cytokine Array C3 by commissioning Shanghai Kangcheng Biotechnology Co., LTD. Mouse cytokines ELISA kits (CXCL1, G-CSF, GM-CSF and CCL2) were used per the manufacturer’s recommendations. For cytokine determination in mouse lung tissue, the left lower lobe lung tissues (about 100 mg/ mouse) were taken, and 500 µl PBS was added. The tissues were mechanically grinded in a high-speed low-temperature tissue grinder (Wuhan, China), followed by centrifuging at 10,000 rmp for 10 min. The supernatant was taken and the protein concentration was determined by BCA (Butyleyanoacrylate) method.

### MDSCs migration assay

Migration assays utilized a 24-well plate Boyden chamber assay with 8 μm pore insert. The purified MDSCs were seeded into the upper compartment of the insert (2 × 10^5^/250µl/well). The inserts were placed in the lower chamber. Subsequently, 500 µl of the medium (RPMI 1640 medium supplemented with 0.5% FBS, 25 mM HEPES, pH 7.4) containing exosomes (50 µg/ml) or lung tissue extract (50 µg/ml) or BMDMs culture supernatant (1:1) were added to the lower chamber. For migration assays, CXCR2 or CCR2 signaling small molecule inhibitor, SB265610 or RS504393, was used at a final concentration of 1µM or 1.5µM, respectively. The migrated cells in the lower chamber were counted after 16–18 h. Experiments were done in triplicates.

### Histology and lung metastases analysis

Lung tissues from the left upper lobe of mice were taken and fixed in 4% paraformaldehyde and embedded in paraffin. Five-micrometre sections were stained with hematoxylin & eosin (HE). Numbers of lung metastases were quantified on seven hematoxylin and eosin-stained representative sections. The area of each lung metastases was measured and divided into two groups according to their size: small metastases with an area less than 0.4 mm^2^ and large metastases with an area greater than 0.4 mm^2^, as described previously [31].

### Western blot

For protein detection, cells supernatant was separated with RIPA buffer (Sigma). 50 µg of protein was separated using a 10 or 12% SDS-PAGE gel. The proteins were transferred onto PVDF membrane (Bio Rad Laboratories) using semi dry Trans-Blot (Bio Rad Laboratories). The primary antibodies were incubated with the membrane overnight at 4 °C. Subsequently, blots were washed and incubated with appropriate secondary antibodies (Beyotime, Haimen, China) for 1 h at room temperature. Images were obtained using a chemiluminescence (ECL) detection system (ProteinSimple, San Jose, CA). Quantified band intensities were normalized using β-actin protein. Blots were scanned with a Tanon imaging system (5200, Shanghai, China). Primary antibodies against Vimentin, E-cadherin, CD9, TSG101, CXCR2, CCR2 were purchased from ABclonal (Wu Han, China).

### Inhibition of T cell function by MDSCs

For T cell proliferation assay, **s**plenocytes from normal BALB/c mice were placed in triplicates into a U-bottom 96-well plates (1 × 10^5^) with or without the presence 10 µg/ml ConA and 10 µg/ml mrIL-2. The splenocytes were co-cultured with purified MDSCs for 68 h and then MTS (15 µl/well) was added for further culture for 4 h. The OD570 value of cell culture supernatant was detected by microplate analyzer.

For CTL killing assay, **s**plenocytes from 4T1 tumor-bearing mice were seeded in 24-well plates with complete RPMI 1640 medium containing recombinant mIL-2 at 10 µg/ml and antigen (from 4T1 cell lysate, 50 µg/ml), and the cultures were incubated for 6 d at 37℃ to induce CTL differentiation. During the incubation period, fresh medium was changed every 48 h. Subsequently, the CTL cells, the purified MDSCs and 4T1 cells were plated in complete RPMI 1640 medium in round bottom 96-well plates in the ratio of 5:1:1. The cultures were incubated for 16 h at 37℃. Then cultured supernatant and suspended cells were removed, the cells were digested with trypsin, and the number of dead cells was detected by flow cytometry after 7-AAD labeling.

4T1 cells co-cultured with MDSCs at the ratio of 10:1 and the representative pictures were shown at 0 and 24 h after wound scratches were made. Migration rates were calculated by measuring the width of the wound scratches, the experiment was repeated three times.

### Cell scratch test

When 4T1 cells seeded in 12-well plate reached a confluent state, a single scratch was made using a sterile 10 µl pipette tip. The cells were then incubated with FBS-free culture medium alone or containing M-MDSCs or PMN-MDSCs (10^5^/well). Images of the scratches were captured at 0, 24 h with Olympus inverted microscope. The width of the scratch was analyzed using the Olympus CellSens Dimension software. Cell mobility was calculated according to the following formula. Mobility = (initial scratch width-current scratch width)/initial scratch width /2 × 100%.

### Differentially expressed genes and enrichment analyses

NCBI GEO database (GSE62817) was used to screen out the differentially expressed genes (DEGs) between normal lung tissues and lung tissues from 4T1 mice with lung metastasis. To identify DEGs associated pathways and function annotations, Gene Ontology (GO) and the Kyoto Encyclopedia of Genes and Genomes (KEGG) enrichment analyses were conducted by DAVID online database (Huang, Sherman & Lempicki, 2009a; Huang, Sherman & Lempicki, 2009b) (DAVID; https://david.ncifcrf.gov).

### Exosome isolation, characterization, and analyses

Isolation of exosomes for antibody array and all other experiments was done by ultracentrifugation, as described previously [25]. 4T1 cells were cultured in complete RPMI 1640 medium for 24 h and at which time the cells confluences rate was about 90%. The cells were irradiated at the dose of 0 or 20 Gy, respectively. Then, the cells were cultured in serum-free RPMI medium for 72 h and the culture medium was collected and centrifuged at 800* g* for 15 min to remove lifted cells and cell debris, and additional 10,000* g* for 30 min to remove microvesicles. Exosomes were then harvested by centrifugation of the supernatant at 100,000 g for 120 min (Beckman Ti70). The exosomes pellet was resuspended in 100 µl of PBS. The morphology and particle size of exosomes were verified by electron microscopy. Western blotting analysis detected rich expression of exosomal specific markers (CD9 and TSG101).

### Exosome labeling, uptaking and treatment

The purified exosomes (50 µg) were labeled using PKH26 membrane fluorescently dye (Sigma) for 20 min at 37 °C. Labeled exosomes were washed in 20 ml of PBS, collected by ultracentrifugation to remove excessive PKH26, and resuspended in PBS. In experiments of exosomes uptake, the BMDMs were cultured with complete RPMI 1640 medium containing labeled exosomes (20 µg/ml) for 7 h and then the cells were harvested, washed with PBS, and analyzed by fluorescence microscope (Nikon, Japan).

For the experiment of stimulating BMDMs to produce cytokines by exosomes, BMDMs were treated with exosomes at a final concentration of 50ug/mL for 24 h. Then, culture supernatant was removed, washed with PBS, and fresh RPMI 1640 complete medium was added and additional culture for 48 h. The supernatant was collected for ELISA experiment.

For the experiment of treating normal mice with exosomes, 5 µg exosomes in 50 µl PBS were injected into the tail vein every other day for 3 weeks. PBS was used as a control. On the second day after the end of exosomes injection, half of the mice (6 mice/group) were sacrificed after anesthesia, and the frequency of MDSCs was analyzed by flow cytometry. The other half of the mice (6 mice/group) were injected with 1 × 10^5^ 4T1 cells through the tail vein and sacrificed 10 days later to observe lung tumor nodules through pathological sections.

### Statistical analysis

Error bars in graphical data represent means ± s.e.m. Statistical significance was determined using a two-tailed Student’s t-test or ANOVA. Statistical analyses were performed using GraphPad Prism software. *P* values were considered statistically significant when p < 0.05 (*p < 0.05; **p < 0.01; and ***p < 0.001).

## Results

### Local radiotherapy increased the number of small metastatic nodules and M-MDSCs in lung although primary tumor progress delayed

**Fig. 1:**
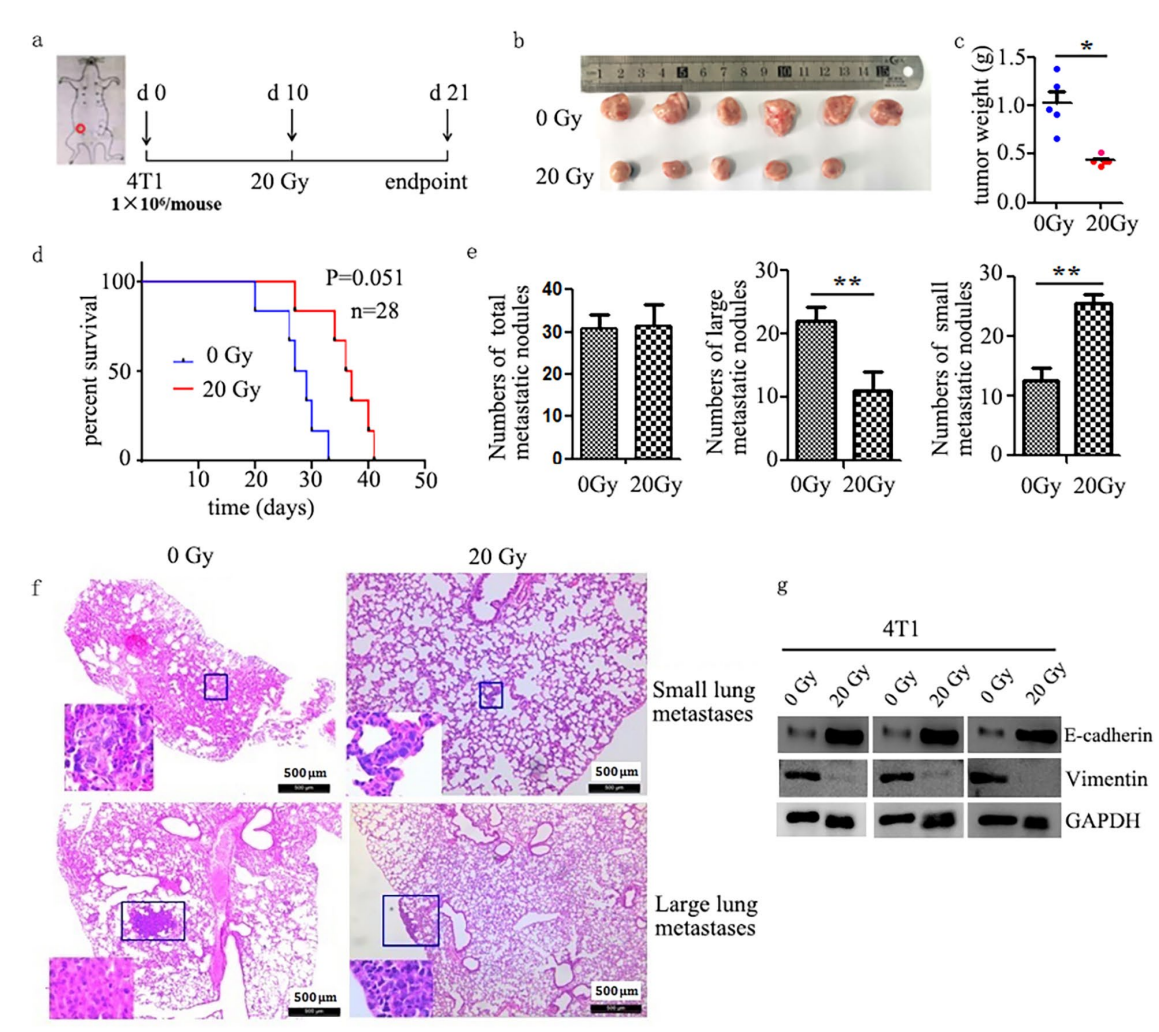
Local X-ray irradiation changed the metastatic nodule numbers in lung of 4T1 tumor-bearing BALB/c mice. **a** Schematic representation of tumor development, and treatment timeline. **b** The tumor volume, **c** tumor weight of tumor-bearing mice in different groups was shown (*P < 0.05). **d** Survival time of tumor-bearing mice in different groups. **e** Number of lung metastatic nodules were counted based on H&E-stained slides (*P < 0.05). The experiment was repeated three times with 6 mice in each group. **f** representative images of metastatic nodules. Images are shown at 4 x magnification. Tumor sections from all 18 mice/group were examined. **g** Vimentin or E-Cadherin expression of tumors from IR or non-IR mice were confirmed by western blotting

It was reported that 4T1 tumors showed recurrence or distal metastasis by recruitment of circulating breast cancer cells post-radiotherapy with a single dose of 20 Gy, and the tumor-infiltrated MDSCs promoted tumor growth and invasion by participating in the formation of pre-metastatic niches, angiogenesis, and immunosuppressive activity [32, 33]. To better understand the effect of local radiotherapy on lung metastasis of tumor cells and whether M-MDSCs are involved, we prepared the 4T1 tumor-bearing mice, which is a typical animal model of basal-like breast cancer and is prone to lung metastasis, and local irradiated the mice with a single dose of 20 Gy X-ray. The data showed that the primary tumors weight was significantly reduced and the survival time of mice also tended to be prolonged (Fig. 1a-d). In addition, microscopic analysis of 4T1 metastases bearing lungs showed that metastatic nodules were observed in 10 and 7 of 18 sections in the untreated or radiotherapy groups, respectively. After radiotherapy, although the total number of nodules did not change, the number of small nodules, which area of each was < 0.4 mm^2^, increased significantly, while the number of large metastatic nodules (their sizes were ≥ 0.4 mm^2^) markedly decreased (Fig. 1e, f). In fact, the mobility of 4T1 cells decreased after 20 Gy X-ray irradiation, as evidenced by lower vimentin and higher E-cadherin expression levels in irradiated 4T1 cells (Fig. 1g).

**Fig. 2:**
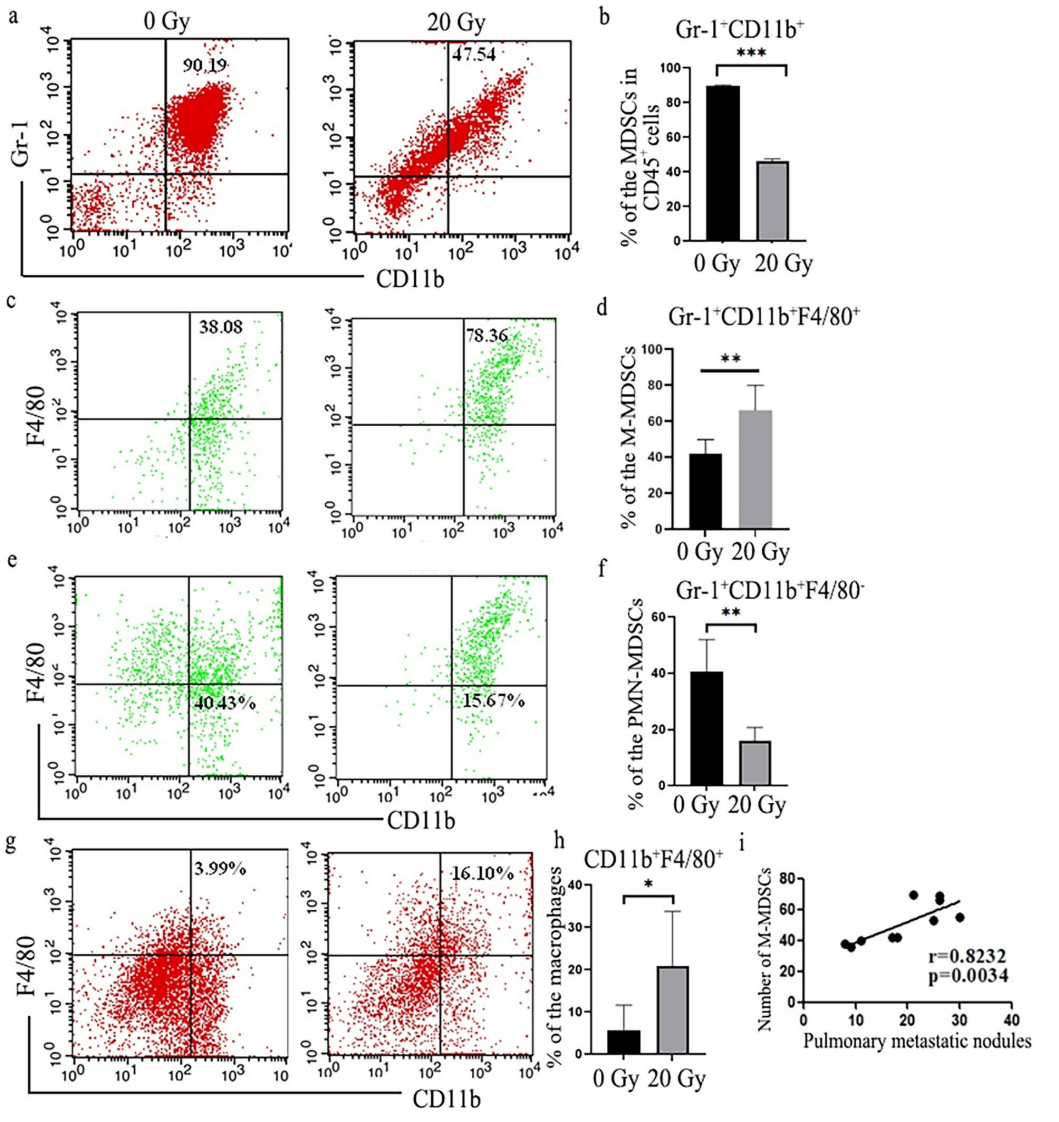
Radiotherapy changed MDSCs subsets distribution in lung of BALB/c mice bearing 4T1 tumor. **a** and **b** Local radiotherapy changed macrophages and MDSCs infiltration of lung in 4T1 tumor-bearing BALB/c mice. The representative flow cytometry data was shown. **c, d, e** and **f** Local X-ray irradiation induced significantly expansion of M-MDSCs, and reduction of PMN-MDSCs, respectively. **g** and **h** Local X-ray irradiation decreased macrophages infiltration. **i** The infiltration number of M-MDSCs in lung tissues was positively correlated with the number of pulmonary nodules

To investigate whether MDSCs and TAM were involved in formation of the metastatic nodules of lung, we next detected the frequency of MDSCs and macrophages in lung of 4T1 tumor-bearing mice. Flow cytometry analysis showed, on day 21 of tumor inoculation, approximately 90% of the mononuclear cells in lung tissue were CD11b^+^Gr-1^+^ MDSCs and local radiotherapy significantly reduced the total MDSCs in the lung. On further analysis showed that radiotherapy decreased the frequency of PMN-MDSCs (CD11b^+^Gr-1^+^F4/80^−^) and increased M-MDSCs (CD11b^+^Gr-1^+^F4/80^+^) recruitment in lung (Fig. 2a-e). Macrophages (Gr-1^−^CD11b^+^F4/80^+^) were less distributed in the lung tissue of 4T1 tumor-bearing mice, and its frequency showed a bit increase post-radiotherapy (Fig. 2f, g). The infiltration number of M-MDSCs in lung tissue had a positively correlation with the number of pulmonary nodules (Fig. 2h). Taken together, the data suggested that the increased frequency of M-MDSCs may be important for the formation of the premetastatic niche in lung.

### The infiltration of MDSCs in the lung of 4T1 tumor-bearing mice may be related to CXCR2 and CCR2 signals

In a set of 4T1 murine breast cancer samples from publicly available datasets from the NCBI GEO database (GSE62817, including 5 normal lung tissues and 4 metastatic samples), we examined 230 differentially expressed genes, 13 of which were 2.5 times higher in lung tissue of 4T1 tumor-bearing mice with lung metastasis than in normal BALB/c mice. David enrichment analysis showed that cytokines and cytokine receptors had the highest correlation with lung metastasis of 4T1 cells. Based on the results of differential genes and enrichment analysis, we concluded that CXCR2 may be one of the key molecules for the recruitment of MDSCs in the lung of 4T1 mice (SFig. 1a, b). Recent studies have shown that in addition to CXCR2 signaling pathway, CCR2 could also participate in the progression of tumors by mediating the recruitment of MDSCs [34, 35]. Then, we analyzed the expression of CXCR2 and CCR2 on M-MDSCs and PMN-MDSCs, which were isolated from lung tissues of 4T1 tumor-bearing mice. The results showed that there was no significant difference in the expression of CXCR2 on M-MDSCs and PMN-MDSCs in lung of tumor-bearing mice. In fact, CCR2 expression was higher on M-MDSCs than on PMN-MDSCs. And both CXCR2 and CCR2 are positively correlated with CD11b expression in human basal-like triple negative breast cancer (SFig. 2a-g). Collectively, MDSCs infiltrated in the lung of 4T1 tumor-bearing mice may be associated with the formation of the pre-metastatic niche and lung metastasis, and CXCR2 and CCR2 might be involved in the recruitment of MDSCs.

### Radiation promoted release of exosomes to induce the recruitment of MDSCs for facilitating the colonization and dissemination of tumor cells

**Fig. 3:**
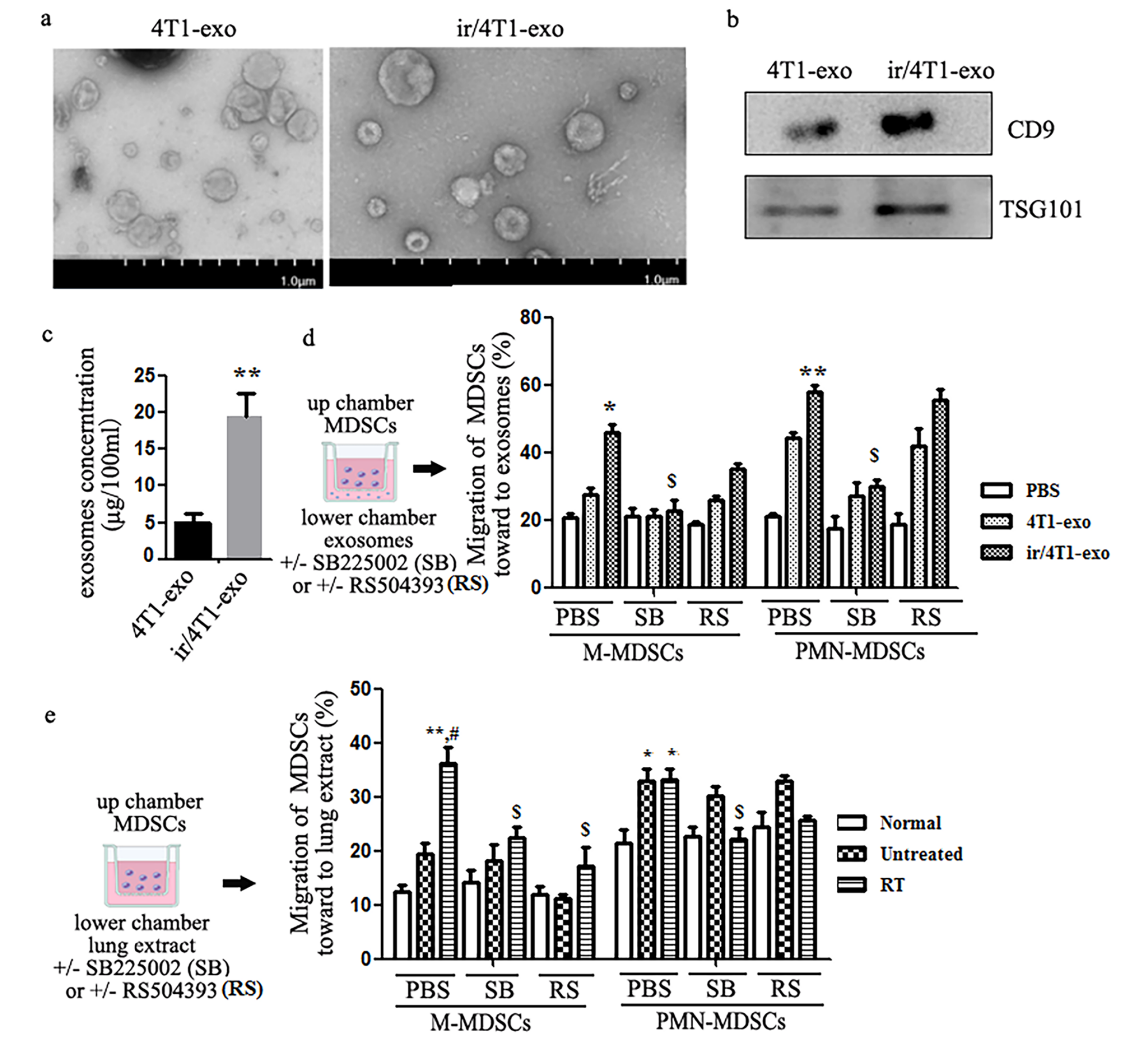
Exosomes released from X-ray-irradiated 4T1 cells promote migration of MDSCs. **a, b** Morphology and chrematistic identification of exosomes. **a** Morphology and size of exosomes were observed by electron microscopy, **b** Western blot. **c** 20 Gy X-ray enhanced exosomes release (The results were repeated three times). **d**In vitro chemotaxis assay of MDSCs towards exosomes derived from 4T1 cells or X-ray irradiated 4T1 cells. Treatment with CXCR2 inhibitor SB225002 or CCR2 inhibitor RS504393 to inhibit CXCR2 or CCR2 signal was performed in MDSCs. Results are expressed as percentage of control (PBS) ± SEM. *p < 0.05, **p < 0.001 vs. PBS; $ *p* < 0.05, $$*p* < 0.01 vs. ir/4T1-exo

Previous studies have demonstrated that tumor cells could recruit myeloid cells to form a premetastatic niche by releasing exosomes [24, 36]. To test the hypothesis that irradiated tumor cell-derived exosomes could impact on MDSCs recruitment and as well as tumor cells colonization in lung, exosomes were isolated from culture supernatants of irradiated (ir/4T1-exo) or non-irradiated 4T1 cells (4T1-exo). As shown in Fig. 3a-c, the exosomes were 100-200 nm in size and expressed CD9 and TSG101 that was consistent with others previous observations [25], and 20 Gy radiation could improve the release of exosomes. Then, we detected the chemotactic effect of the exosomes on MDSCs cells with the method of transwell in vitro. The data showed that 4T1-exo and ir/4T1-exo had chemotaxis on both M-MDSCs and PMN-MDSCs, and the effect of ir/4T1-exo was more obvious. Exosomes-mediated MDSCs’ migration could be reversed by SB265610 (400nM) but not by RS504393, the CCR2 inhibitor (1µM). The results indicated that only CXCL1/CXCR2 signal involved in exosomes-mediated MDSCs’ migration in vitro. Interestingly, compared with the untreated of tumor-bearing mice, the lung tissue extracts of mice followed radiotherapy was more beneficial to M-MDSCs migration, which was consistent to the results of radiotherapy could facilitate the recruitment of M-MDSCs to the lung. Both RS504393 and SB265610 could significantly depress lung tissue extract-mediated M-MDSCs’ migration (Fig. 3d, e). The results showed that CXCR2 and CCR2 signals were involved in the chemotaxis of M-MDSCs mediated by lung extract.

**Fig. 4:**
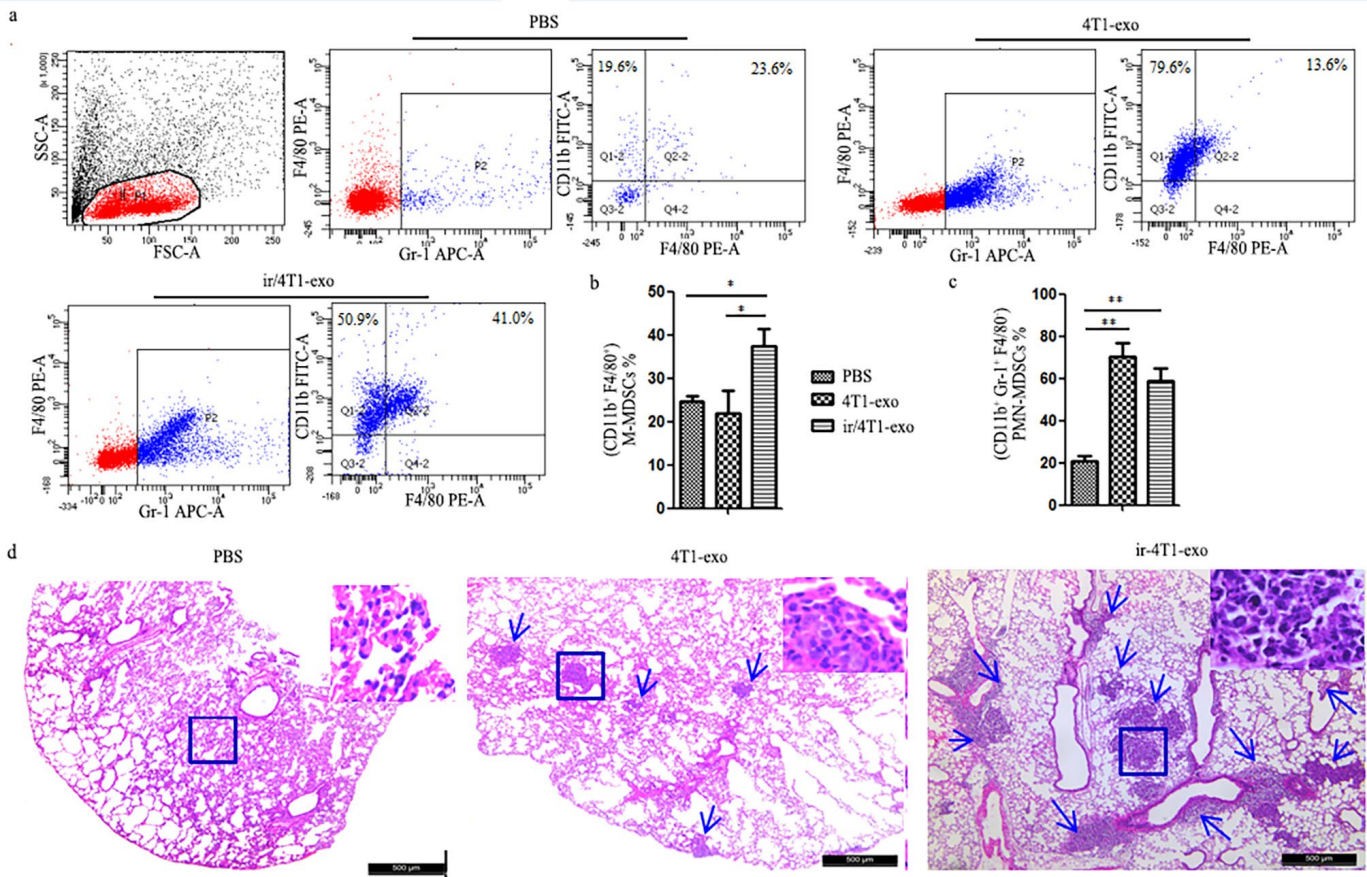
Irradiated 4T1-derived exosomes (ir/4T1-exo) promoted the recruitment of M-MDSCs and colonization circulating 4T1 cells in normal mouse lungs. **a-c** Normal BALB/c mice were injected exosomes (4T1-exo or ir/4T1-exo) every other day for 3 weeks, M-MDSC or PMN-MDSCs frequency was analyzed by FCM (*p < 0.05, **p < 0.01). The representative flow cytometry data was shown. **d** On the second day after exosome injection, 5x10^4^/mouse 4T1 cells were inoculated via tail vein. Representative images of lung metastatic nodules were shown. Images are shown at 4 x magnification. Tumor sections from all 18 mice/group were examined

In order to clarify the effect of exosomes on M-MDSCs recruitment and CTCs colonization in lung, we injected exosomes into normal BALB/c mice every other day for 3 weeks through the tail vein. As expected, the number of M-MDSCs or PMN-MDSCs in the lungs was significantly higher than that of mice injected with PBS. Importantly, ir/4T1-exo could recruit more M-MDSCs than PMN-MDSCs into the lungs. Moreover, the mice injected with exosomes allowed more circulating 4T1 to colonize and form nodules in the lungs (Fig. 4).

**Fig. 5:**
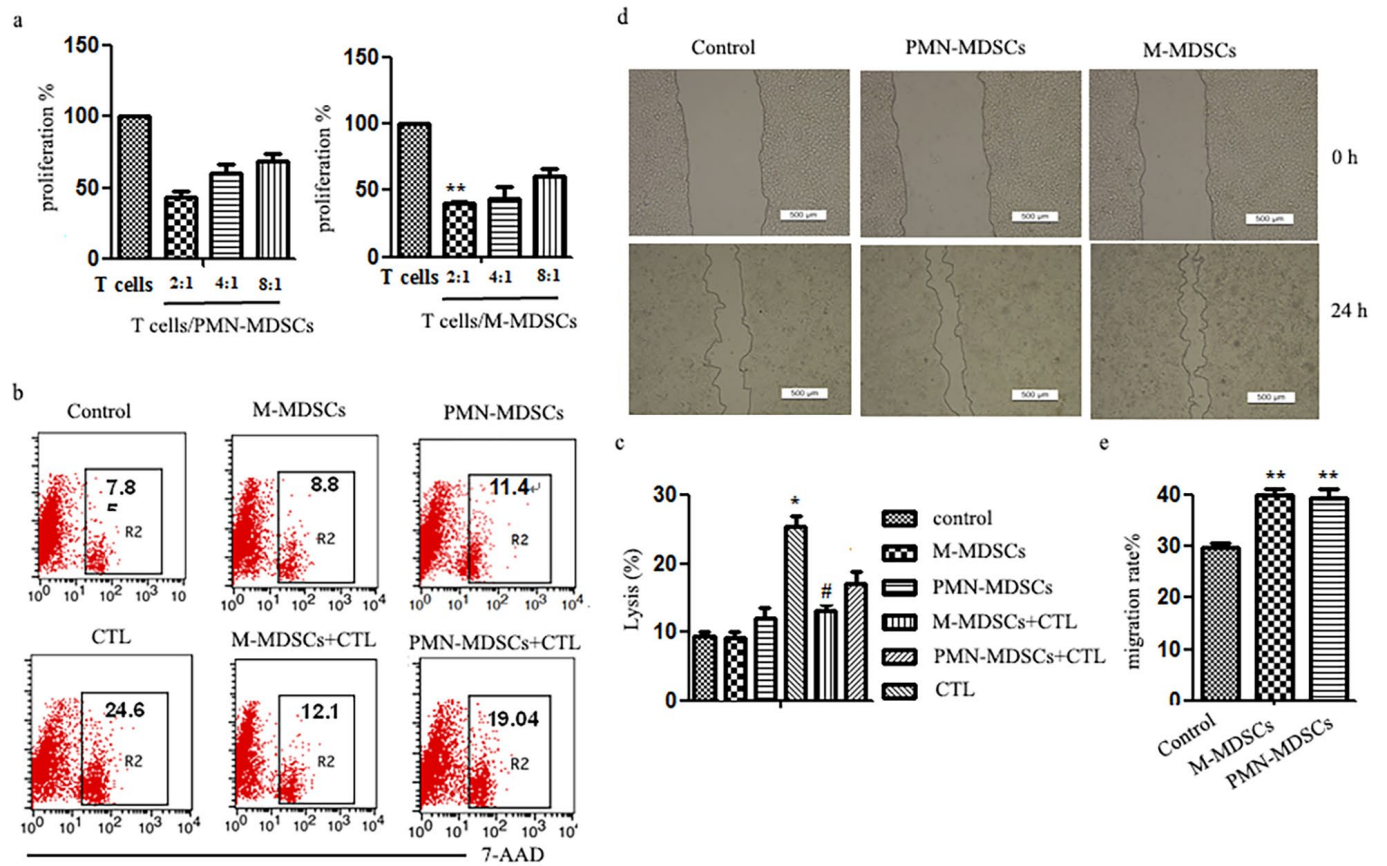
The effect of MDSCs on T cell function and 4T1 cell migration **a** Normal mouse spleen mononuclear cells were mixed with purified MDSCs at the rate of 2:1, 4:1, 8:1, respectively, Con A (10 µg/ml) and mrIL-2 (10 µg/ml) were added and then co-cultured for 68 h. The number of viable cells was detected by MTS method. Compared with T cell, **p < 0.01. **b** Spleen mononuclear cells of 4T1 tumor-bearing mice were stimulated with 4T1 cell lysate and mrIL-2 (10 µg/ml) for 5d and then co-cultured with 4T1 cells with or without MDSCs at the ratio of 10:1:1 for 24 h. The dead 4T1 cells were determined using flow cytometry. **c** The migration distance was measured to analyze the migration ability of 4T1 cells which were impacted on by M-MDSCs or PMN-MDSCs for 24 h. Compared for control, **p < 0.01. Migration rates were calculated by measuring the width of the wound scratches, The experiment was repeated three times.

MDSCs are a chief component of immunosuppressive networks and can promote tumor progression by different ways. Next, we examined the effect of MDSCs from lung of 4T1 tumor-bearing mice on T cell function and 4T1 cell migration. Compared with control, M-MDSCs could obviously inhibit T cells proliferation and CTL killing activity (Fig. 5). Both M-MDSCs and PMN-MDSCs could significantly facilitate the migration of 4T1 cells, and there was no difference between them.

Taken together, although local radiotherapy significantly delayed primary tumor growth tumor migration, irradiation could increase exosomes release. The ir/4T1-exo had a strong ability to promote the entry of M-MDSCs into the lung to form pre-metastatic niche, which increase the risk of 4T1 cells spread in the lung by promoting 4T1 cell migration and inhibiting T cell function.

### G-CSF, GM-CSF and CXCL1-rich exosomes released from irradiated 4T1 cells promote CCL2 production associated with M-MDSCs infiltration in lung

**Fig. 6:**
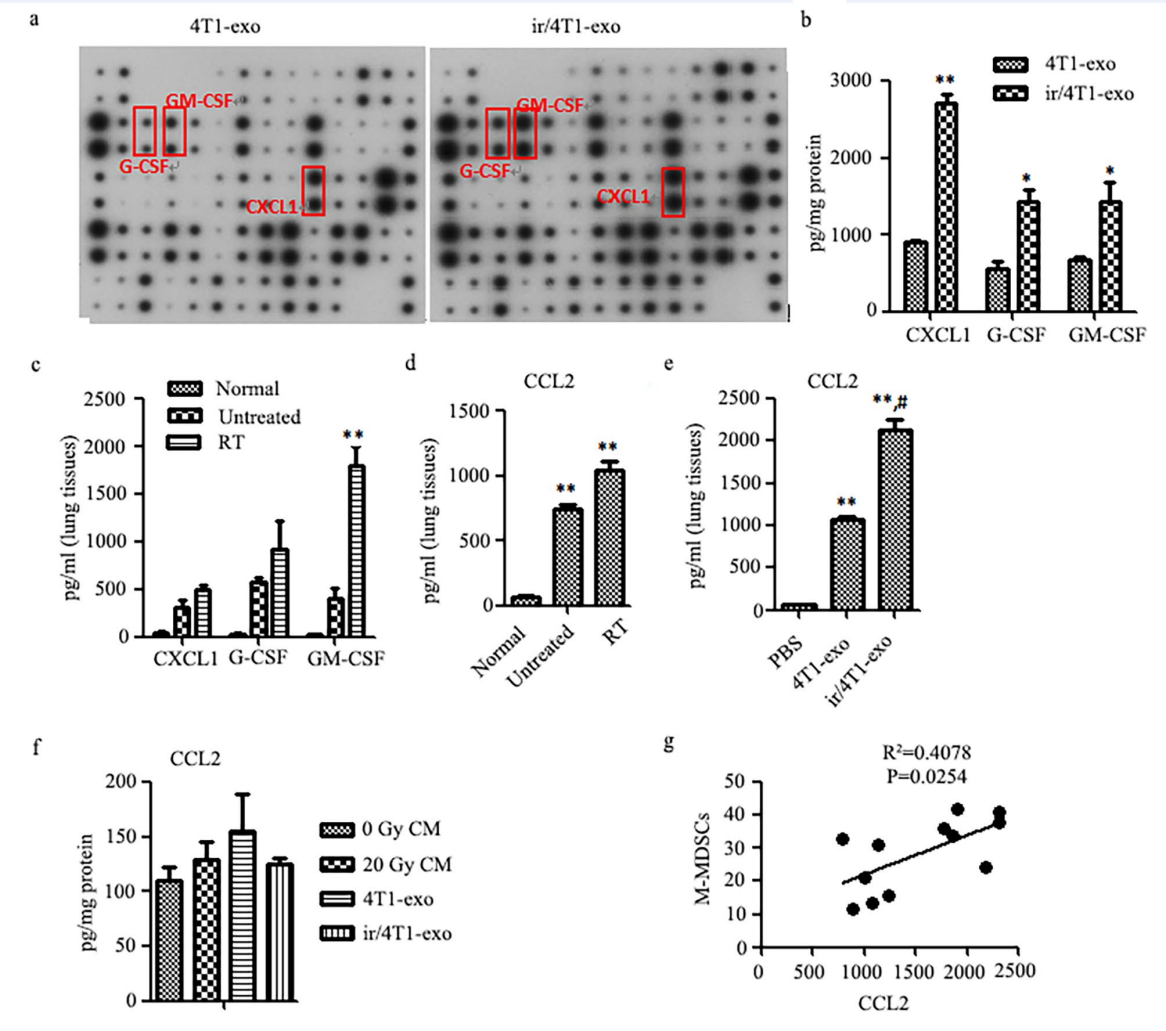
X-ray irradiation enhances G-CSF, CXCL1 and GM-CSF production by 4T1 tumor cells. **a** The cytokine/chemokine profile in the 4T1-exo or ir/4T1-exo were examined by mouse cytokine array. Pixel density in each spot of the array was determined using ImageJ. **b** The content of G-CSF, CXCL1 and GM-CSF in the exosomes was measured by ELISA. Compared with 4T1-exo, *p < 0.05, **p < 0.01. **c** and **d** The level of G-CSF, CXCL1, GM-CSF and CCL2 in lung tissues of 4T1 tumor-bearing mice before or after radiotherapy (n = 3 per group) was detected with the method of ELISA. Compared with normal and untreated mice, **p < 0.01. e CCL2 level in lung extracts of normal BALB/c mice after receiving exosomes injection. Compared with injection of PBS or 4T1-exo, **p < 0.01 or # *p* < 0.05, respectively. **f** CCL2 level in cell culture supernatant or exosomes derived from 4T1 or irradiated-4T1 cells. **g** The level of CCL2 in mouse lung tissue extracts was positively correlated with the number of M-MDSCs.

4T1 murine tumor cells secreted higher levels of inflammatory cytokines, such as IL6, IL8, RANTES, G-CSF, GM-CSF, IL-12 [37], which can impact on MDSCs differentiation or cancer cells metastasis. To explore whether cytokines released by the irradiated 4T1 cells could alter the MDSCs frequency in lung through exosomes and subsequently affect lung metastasis of tumor, we used a cytokine antibody array and ELISA to determine some cytokines/chemokines in exosomes. The results showed that X-ray irradiation increased the levels of GM-CSF, G-CSF and CXCL1 in exosomes secreted by 4T1 cells (Fig. 6a, b).

G-CSF and GM-CSF are critical factors in accumulation of MDSCs by regulating bone marrow cell differentiation, while CXCL1 had chemotactic effect on MDSCs expressing CXCR2 [38, 39]. Considering that lung tissue extracts from mice post-radiotherapy could selectively promote the recruitment of M-MDSCs, we determined these cytokines and as well CCL2 in lung tissues. The data showed that compared with untreated mice, the content of GM-CSF and CCL2 remarkably increased in lung tissues of 4T1 tumor-bearing mice post-radiotherapy. Further, high levels of CCL2 were also detected in lung tissue from normal mice that were injected with ir/4T1-exo. However, the content of CCL2 in 4T1 cell culture supernatant or exosomes was low (Fig. 6c, f). These results suggested that 4T1 cells had the capacity to produce CCL2 at a low level, and the main source of CCL2 in lung tissues of 4T1 tumor-bearing mice was lung stromal cells. Furthermore, the content of CCL2 in lung tissue was positively correlated with the number of M-MDSCs (Fig. 6g).

### Lung macrophages promoted the production of CCL2 through uptaking exosomes and autocrine GM-CSF

**Fig. 7:**
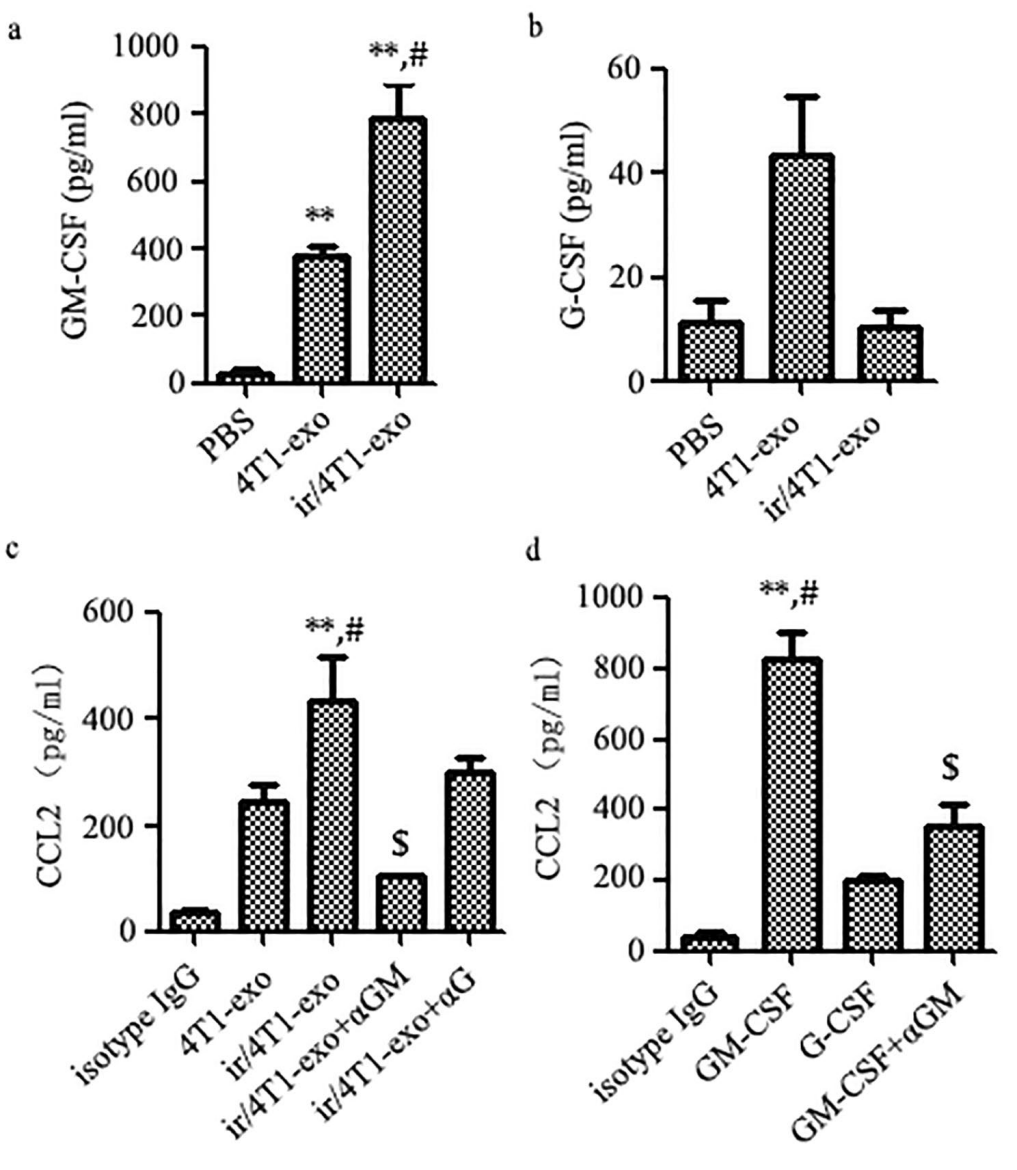
ir/4T1-exo treatment enhances GM-CSF, CCL2 production by BMDMs. **a** GM-CSF **b** G-CSF level in the culture supernatant of exosomes-treated BMDMs was measured by ELISA. Compared with PBS, **p < 0.01; compared with 4T1-exo, *# p <* 0.05. **c** CCL2 level in the culture supernatant of exosomes-treated BMDMs with or without antibody of anti-GM-CF or anti-G-CSF. Compared with isotype IgG, *p < 0.05; **p < 0.01; compared with 4T1-exo, *# p <* 0.05; compared with ir/4T1-exo, $ *p* < 0.05. **d** CCL2 production by BMDMs treated with GM-CSF or G-CSF with or without antibody of anti-GM-CF. Compared with isotype IgG, **p < 0.01; compared with GM-CSF, $ *p* < 0.05

**Fig. 8:**
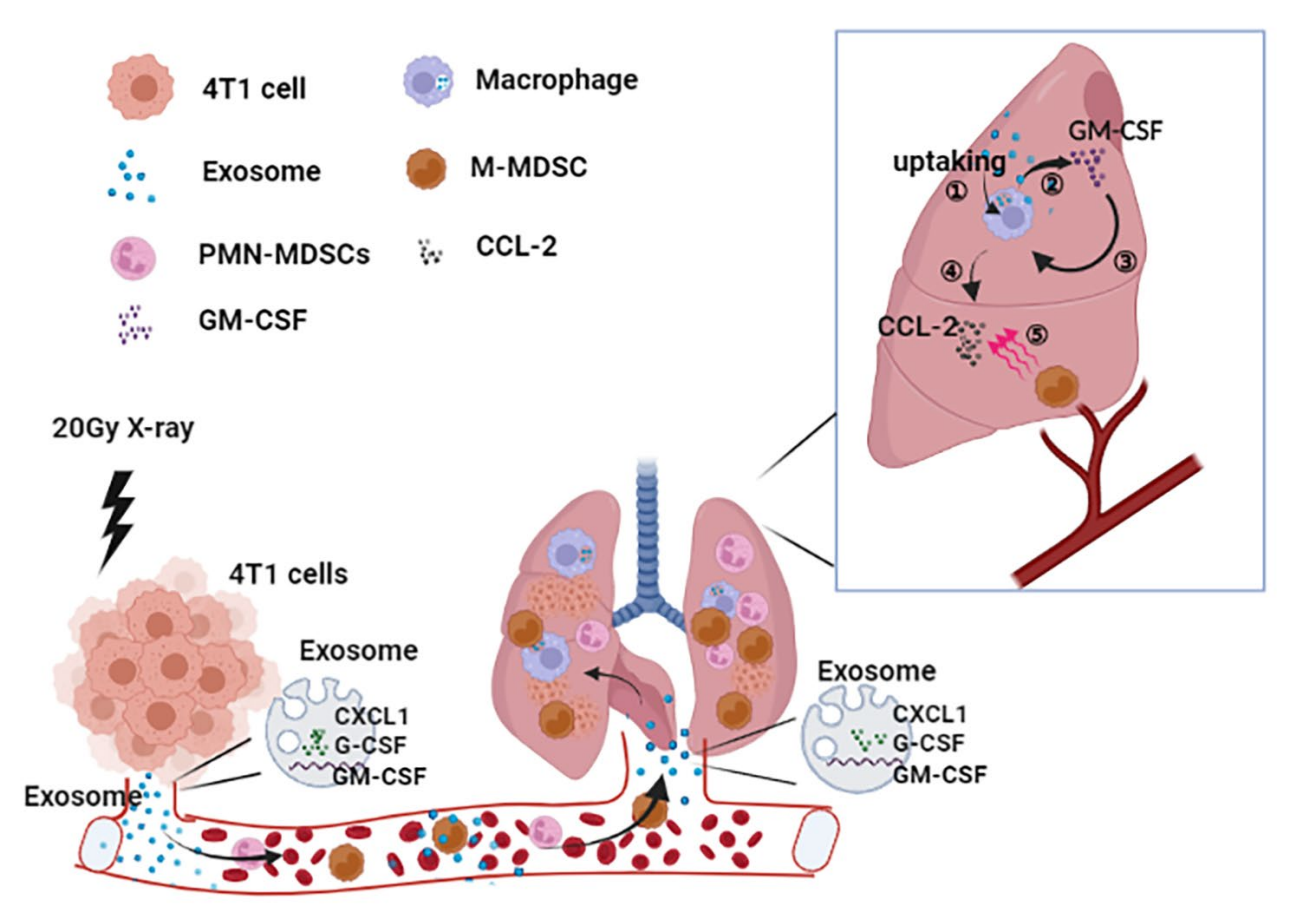
Radiotherapy promoting the recruitment of M-MDSCs to the lung of 4T1 tumor-bearing mice via CXCL1-CXCR2 and CCL2-CCR2 signals

Taking into account that radiotherapy could increase the production of GM-CSF and CCL2 in lung of tumor-bearing mice, and that GM-CSF is an important mediator in inducing the production of CCL2 [40, 41], we investigated whether G-CSF or GM-CSF could induce macrophages to produce CCL2. With this purpose, we first treated mouse bone marrow cells by M-CSF to obtain mouse BMDMs (SFig. 2), then, the BMDMs were treated with 4T1-exo (50 µg/ml), ir/4T1-exo (50 µg/ml), G-CSF (10ng/ml) or GM-CSF (25ng/ml), respectively. The results showed that ir/4T1-exo could induce GM-CSF and CCL2, but not G-CSF production (Fig. 7a, c). Concurrently, ir/4T1-exo and GM-CSF, but not G-CSF could significantly stimulate BMDMs to produce CCL2. And the αGM-CSF but not αG-CSF could almost completely abrogate exosomes-induced CCL2 production by BMDMs (Fig. 7c, d). As expected, BMDM culture medium pre-treated with ir/4T1-exo showed selective potentiation of chemotaxis for M-MDSCs. Further, RS504393 could remarkably decrease the migration of M-MDSCs (SFig. 3). These data suggested that GM-CSF-rich exosomes could stimulate macrophage producing more GM-CSF, which in turn further promoted CCL2 release in an autocrine manner (Fig. 8). Taken together, these results further suggest that radiotherapy could induce an undesired effect on recruiting M-MDSCs toward lung to form immunosuppressive premetastatic niches via exosomes derived from residual tumor cells that survive from radiotherapy.

Local RT promotes the release of exosomes rich in G-CSF and CXCL1 from 4T1 tumors. Exosomes enter the lungs through the blood circulation. On the one hand, CXCL1 can directly involve in the chemotaxis CXCR2^+^ M-MDSCs through the CXCL1-CXCR2 axis. On the other hand, exosomes are taken up by lung macrophages and induced to produce GM-CSF and CCL2, respectively. Furthermore, GM-CSF can further induce macrophages to release CCL2 through autocrine secretion, and finally, CCR2^+^ M-MDSCs enters the lung through CCL2-CCR2.

## Discussion

Accumulating evidence indicates that radiotherapy treatment induces not only systemic anti-tumor immunity [42, 43] by increasing NK and CD8^+^ CTL infiltration and reducing the level of invasive regulatory T cells (Treg) [44], but also tumor recurrence and metastasis, by “awakening” cancer stem cells or improving myeloid cells recruitment [45–47]. Tumor self-seeding and the formation of distal pre-metastatic niche are the characters for triple negative breast cancers (TNBC) metastasis [14, 48, 49]. Mouse breast cancer 4T1 cells are the classic model of metastatic TNBC with mesenchymal phenotype [37, 50] and it has similar characteristics with human basal sample TNBC [51]. Murine 4T1 tumor-bearing mice usually develop lung metastases on about day 21 ~ day 28. However, the effect of radiotherapy on the formation of premetastatic niches in distant organs that support the colonization of cancer cells remains poorly understood. Compared with the tumor-bearing mice without radiotherapy, although local radiotherapy did not change total number of lung metastatic nodules, the number of small nodules significantly increased. In other words, radiotherapy does not exclude the risk of lung metastasis. We believed that, due to local radiotherapy did not increase the migration potential of 4T1 cells, local lung microenvironment adapted to CTCs colonization following radiotherapy treatment leads to the observed effects.

MDSCs are an important component of pre-metastasis niche, and there are a large number of MDSCs in lung of 4T1 tumor-bearing mice [22, 52, 53]. Previous study has shown that radiotherapy can activate STING/type I interferon pathway of tumor cells to enhance suppressive inflammation in TEM by recruiting myeloid cells in part via the CCR2 pathway [54]. Generally, in tumor-bearing mice model, the number of PMN-MDSCs is more than that of M-MDSCs, but M-MDSCs are found to have a stronger inhibitory effect on T cell response on a per cell basis [55]. In this study, we show that there are a large number of Gr-1^+^CD11b^+^ MDSCs in lung tissues of 4T1 tumor-bearing mice, and the number of Gr-1^+^CD11b^+^F4/80^−^ granulocytic-MDSCs (PMN-MDSCs) is more than that of Gr-1^+^CD11b^+^F4/80^+^ monocytic-MDSCs (M-MDSCs). Importantly, radiotherapy treatment can change the distribution pattern of MDSCs subsets in lung, including increasing the number of M-MDSCs and decreasing the number of PMN-MDSCs. We propose that some mediators produced by tumor cells following radiotherapy change the recruitment status of MDSCs subsets.

Under radiotherapy condition, the driving force of M-MDSC recruitment to the lung should be chemokines and chemotaxis receptors in the first place. CXCR2 and CCR2 are the most important chemokine receptors for chemotactic PMN-MDSCs and M-MDSCs. Indeed, some studies in mouse head and neck cancer and gastric cancer have shown PMN-MDSCs and M-MDSCs can be recruited to spleens or tumors of tumor-bearing mice by CXCR2 or CCR2 signaling, respectively [56, 57]. Other study also confirme that expression level of CXCR2 on PMN-MDSCs within tumors is higher than that on M-MDSCs [30]. However, we do not observe the obviously differences of CXCR2 expression between PMN-MDSCs and M-MDSCs from lung of 4T1 tumor-bearing mouse. The expression of CXCR2 on M-MDSCs may change with different spatial locations, such as tumor in situ and lung metastasis. Previous evidence has revealed that the CCL2/CCR2 axis can not only recruit monocytes [34], but also exert chemotactic effect on M-MDSCs [58, 59]. Our results show CCR2 is more highly expressed on M-MDSCs than PMN-MDSCs, and CCR2 or CXCR2 expression is positively correlated with CD11b^+^ cell infiltration in basal breast cancer. Collectively, our data further indicate that CXCR2 and CCR2 are important signals for M-MDSCs accumulating to lung following radiotherapy treatment.

Actually, it has been demonstrated that tumor-derived exosomes can lead to pre-metastatic niches formation in distal organs by recruiting myeloid cells [36]. Additionally, murine breast cancer-derived exosomes enter the lung primarily and are associated with pre-lung metastases niche formation [60]. However, it is unclear what links exosomes from tumor cells following irradiation to modulation of MDSCs in lung. Here, we demonstrate that both 4T1-exo and ir/4T1-exo have the ability to mobilize M-MDSCs and PMN-MDSCs migration in vitro, and CXCR2 signaling may be involved in this process. However, when 4T1-exo and ir/4T1-exo were injected into normal mice by the tail vein, accumulation of M-MDSCs and PMN-MDSCs can be detected in lung. Moreover, ir/4T1-exo has a more obvious selective effect on M-MDSCs recruitment than PMN-MDSCs. Further, when 4T1 cells are injected through the tail vein into normal mice that pretreated with exosomes, more metastatic nodules can be observed in the lungs of mice that received ir/4T1-exo injection. On one hand, the results are related with an increase in M-MDSCs in lung of mice post-radiotherapy. On the other hand, these results also suggest that the entry of exosomes into mice may change the inflammatory microenvironment of lung, leading to more favorable entry of M-MDSCs to form pre-metastatic niche and facilitate CTCs colonization. Additionally, although M-MDSCs and PMN-MDSCs show the similar ability to enhance the migration of 4T1 cells in vitro, M-MDSCs has stronger potential to inhibit the proliferation of T cells and the CTL killing activity against 4T1 cells. These results further indicate that radiotherapy treatment change the distribution of M-MDSCs in lung microenvironment of tumor-bearing mice by releasing exosomes to increase the risk of metastasis.

Tumor-derived exosomes can participate in the recruitment of myeloid cells and the formation of pre-metastasis niche by relying on the cytokines they carry [37]. Moreover, some cytokines, such as CXCL1/CXCR2 axis [61], G-CSF [38, 62], GM-CSF [63] and VEGF-C [64], have been confirmed to be involved in the recruitment of MDSCs in tumor-bearing mice. Knockdown of GM-CSF in tumor cells or blockade it with antibody also delays tumor progression with decreased accumulation of M-MDSC in TME [65]. Therefore, we evaluate some cytokines from exosomes derived from irradiated or non-irradiated 4T1 tumor cells (ir/4T1-exo or 4T1-exo). The data indicate that high level of G-CSF, CXCL1 and GM-CSF, which is thought to be an important player in the differentiation and recruitment of MDSCs, are found in ir/4T1-exo. Moreover, ir/4T1-exo has similar chemotactic effects on both M-MDSCs and PMN-MDSCs. CCR2 has been reported to provide signaling for licensing M-MDSCs migration induced by CCL2 [34, 55]. We observe high levels of GM-CSF and CCL2 in lung tissue extracts from 4T1 tumor-bearing mice after radiotherapy and normal mice injected with exosomes, which showed a chemotactic tendency to M-MDSCs.

Considering the complexity of differentiation and recruitment of MDSCs induced by cytokines, we speculate that the exosomes rich in CXCL1/G-CSF/GM-CSF released by 4T1 cells following radiotherapy can enter the lung, and subsequently, the exosomes may be ingested by macrophages in the lung. Finally, the macrophages stimulated by exosomes to produce high levels of GM-CSF and CCL2. This hypothesis is preliminarily verified by the results that macrophages derived from mice bone marrow can release a higher level of GM-CSF and CCL2 than G-CSF after intaking the ir/4T1-exo. After blocking GM-CSF but not G-CSF with antibody, CCL2 production was significantly blocked. Taken together, these data, along with other studies, provide a potential causal link between tumor-derived inflammatory cytokines carried by exosomes and accumulation of M-MDSCs in radiotherapy setting.

Considering that radiotherapy is widely used in tumor treatment, whether reducing MDSCs migration by inhibiting CXCR2 and CCR2 signaling can promote the therapeutic effect of radiotherapy will be the focus of our subsequent study.

## Electronic supplementary material

Below is the link to the electronic supplementary material.

**Supplementary Table S1** Summary of materials

**Supplementary Figures: SFig.1** Analysis of differentially expressed genes in lung tissue of 4T1 tumor-bearing mice. **a** Differentially expressed genes between lung tissue expression of BALB/c normal mice and 4T1 tumor-bearing mice in the GEO database (GSE62817) were analyzed, and genes with a difference of 2.5 times or more were shown. **b** David enrichment analysis showed that cytokines and cytokine receptors were highly correlated with 4T1 lung metastasis. **SFig. 2** CXCR2 and CCR2 expression in MDSCs. **a** Purity analysis of MDSCs derived from 4T1 tumor-bearing mice. Mononuclear cells from lung tissue of 4T1 tumor-bearing mice were obtained by Ficoll density gradient centrifugation. Then CD11b^+^ cells were obtained from lung mononuclear cells by sorting flow cytometry. Finally, Ly6G^+^ PMN-MDSCs or Ly6C^+^ M-MDSCs were sorted by immunomagnetic beads in CD11b^+^ cells. Purity was analyzed by flow cytometry. **b,c,d** and **e** CXCR2 or CCR2 expression on M-MDSCs/PMN-MDSCs in mononuclear cell of 4T1 tumor-bearing mice lung tissues. **f** and **g** Correlation of CCR2 and CXCR2 with CD11b^+^ myeloid cells infiltration in basal breast cancer was analyzed via the Oncomine database and Tumor Immune Estimation Resource (TIMER) site. CXCR2 and CCR2 showed significant positive correlation with CD11b^+^ myeloid cells. **SFig.3** Conditioned medium (CM) of ir/4T1-exo pretreated BMDMs promotes the migration of M-MDSCs. **a** Bone marrow cells of BALB/c mice were treated with M-CSF for 9d to induce bone morrow derived macrophage (BMDMs) differentiation, then, CD11b^+^F4/80^+^ BMDMs frequency was detected by flow cytometry. **b** Exosomes were isolated from 4T1 cells (4T1-exo) or 20 Gy radiation irradiated 4T1 cells (ir/4T1-exo) using the ultracentrifugation method and labeled with PKH26. Uptake of PKH26-labeled exosomes at 37 °C for 7 h and observed by fluorescence microscope. **c** BMDMs were pretreated with additional 4T1-exo or ir/4T1-exo (50 µg/ml) at 37 °C for 24 h, followed by wash and 48 h culture for CM collection. The migration of M-MDSCs and PMN-MDSCs were measured by transwell. Compared with PBS pretreated CM, * *p* < 0.05; Compared with ir/4T1-exo pretreated CM, # *p* < 0.05.

## Data Availability

Not applicable.
