# Supplementary material for: Local radiotherapy for murine breast cancer increases risk of metastasis by promoting the recruitment of M-MDSCs in lung
Source: Cancer Cell Int. 2023 Jun 2;23:107. doi: 10.1186/s12935-023-02934-6 (PMC10236833; doi:10.1186/s12935-023-02934-6)
Supplement: Supplementary file 1 — Supplementary Table S1 Summary of materials [file 12935_2023_2934_MOESM1_ESM.docx]

**Supplementary Table S1** Summary of materials

| **Materials** | **Manufacturers** | **Cat#** |
| --- | --- | --- |
| Anti-CD11b-488 | BD Biosciences | 128015 |
| Anti-Ly6C-PE | BD Biosciences | 560595 |
| Anti-Ly6G-PE | BD Biosciences | 560592 |
| Anti-PE microbeads | Miltenyi Biotec | 130-048-801 |
| Anti-Gr-1-APC | BD Biosciences | 127605 |
| Anti-F4/80-PE | BioLegend | 101208 |
| Anti-CXCR2-PerCP/Cy5.5 | BioLegend | 149307 |
| Anti-CCR2-APC | BioLegend | 150627 |
| Anti-CD16/CD32 | BD Biosciences | 156603 |
| Mouse Cytokine Array C3 | RayBiotech | AAM-CYT-3-8 |
| CXCL1 ELISA kits | ABclonal | RK00038 |
| G-CSF ELISA kits | ABclonal | RK00048 |
| GM-CSF ELISA kits | ABclonal | RK00049 |
| CCL2 ELISA kits | ABclonal | RK00381 |
| GM-CSF | PeproTech | AF-315-03-20 |
| G-CSF | PeproTech | AF-250-05 |
| M-CSF | PeproTech | AF-315-02-100 |
| Anti-GM-CSF | Abcam | [ab220888](https://www.so.com/link?m=b3wYuBh0A2cSuHBO7DbtevvPI4l60D1Fo%2F2gn3V2uzDwHtcXX3lxOVaPTwgd2tWFI5Qb%2F1ugQEH4nOYZJMl1p37x2f%2BF51EJz9E5nrE1%2BgPs7%2F3t5C9YS2emPDbD79qOzSNPIzz6pSdx1Txu7%2BDM5cvqgH2ivGkKrl%2FPUG7xbmRMjM%2Fe4VwwNFkcXvu0%3D) |
| Anti-G-CSF | [Abcam](https://www.so.com/link?m=b8ijOz%2BW6BbRYDxAzHveMd2E5ZoNTBJTzDpA1D9mGIhRlQQzCM2GoiAnm5xnXwm8MMFi8N%2BgnSoLOMJTtSYcQ3zIWgGyz8WYUrsi288mm20s7t5jIH5yETK3c0QJrNb%2BvrPPRj6ZlcRvfd8ikECsfVN0IIhkRO3Dc6qOTXlSFFs1FvIrDasWAvA%3D%3D) | ab9691 |
| Anti-Vimentin | ABclonal | A19607 |
| Anti-N-cadherin | ABclonal | A0433 |
| Anti-E-cadherin | ABclonal | A20798 |
| Anti-CD9 | ABclonal | A19027 |
| Anti-TSG101 | ABclonal | A5789 |
| Anti-CXCR2 | ABclonal | A2889 |
| Anti-CCR2 | ABclonal | A2855 |
| CXCR2 inhibitor SB265610 | MCE | 211096-49-01 |
| CCR2 inhibitor RS504393 | ChemSrk | [300816-15-3](https://m.chemsrc.com/en/baike/671963.html) |
| RPMI 1640 medium | Thermo Fisher | C11875500BT |
| Anti-CD3-APC | Toscience Biotechnology | 100235 |
| mrIL-2 | PeproTech | AF-212-12-100 |
| MTS | Abcam | 138169-43-4 |
| 7-AAD | AAT Bioquest | [7240-37-1](https://www.medchemexpress.cn/search.html?q=7240-37-1&ft=&fa=&fp=) |
| PKH26 membrane fluorescently dye | Sigma | MINI26 |
| FBS | HyClone, Beijing wobisen technology co., LTD | SH30084.03 |
