# Supplementary material for: Local radiotherapy for murine breast cancer increases risk of metastasis by promoting the recruitment of M-MDSCs in lung
Source: Cancer Cell Int. 2023 Jun 2;23:107. doi: 10.1186/s12935-023-02934-6 (PMC10236833; doi:10.1186/s12935-023-02934-6)
Supplement: Supplementary file 2 — Supplementary Figures: SFig.1 Analysis of differentially expressed genes in lung tissue of 4T1 tumor-bearing mice. a Differentially expressed genes between lung tissue expression of BALB/c normal mice and 4T1 tumor-bearing mice in the GEO database (GSE62817) were analyzed, and genes with a difference of 2.5 times or more were shown. b David enrichment analysis showed that cytokines and cytokine receptors were highly correlated with 4T1 lung metastasis. SFig. 2 CXCR2 and CCR2 expression in MDSCs. a Purity analysis of MDSCs derived from 4T1 tumor-bearing mice. Mononuclear cells from lung tissue of 4T1 tumor-bearing mice were obtained by Ficoll density gradient centrifugation. Then CD11b+ cells were obtained from lung mononuclear cells by sorting flow cytometry. Finally, Ly6G+ PMN-MDSCs or Ly6C+ M-MDSCs were sorted by immunomagnetic beads in CD11b+ cells. Purity was analyzed by flow cytometry. b,c,d and e CXCR2 or CCR2 expression on M-MDSCs/PMN-MDSCs in mononuclear cell of 4T1 tumor-bearing mice lung tissues. f and g Correlation of CCR2 and CXCR2 with CD11b+ myeloid cells infiltration in basal breast cancer was analyzed via the Oncomine database and Tumor Immune Estimation Resource (TIMER) site. CXCR2 and CCR2 showed significant positive correlation with CD11b+ myeloid cells. SFig.3 Conditioned medium (CM) of ir/4T1-exo pretreated BMDMs promotes the migration of M-MDSCs. a Bone marrow cells of BALB/c mice were treated with M-CSF for 9d to induce bone morrow derived macrophage (BMDMs) differentiation, then, CD11b+F4/80+ BMDMs frequency was detected by flow cytometry. b Exosomes were isolated from 4T1 cells (4T1-exo) or 20 Gy radiation irradiated 4T1 cells (ir/4T1-exo) using the ultracentrifugation method and labeled with PKH26. Uptake of PKH26-labeled exosomes at 37 °C for 7 h and observed by fluorescence microscope. c BMDMs were pretreated with additional 4T1-exo or ir/4T1-exo (50 µg/ml) at 37 °C for 24 h, followed by wash and 48 h culture for CM collection. The migrat [file 12935_2023_2934_MOESM2_ESM.docx]

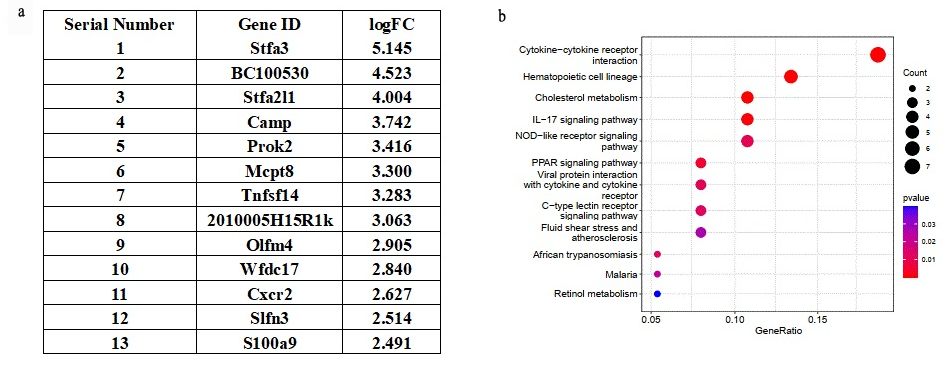


**SFig.1** Analysis of differentially expressed genes in lung tissue of 4T1 tumor-bearing mice. **a** Differentially expressed genes between lung tissue expression of BALB/c normal mice and 4T1 tumor-bearing mice in the GEO database (GSE62817) were analyzed, and genes with a difference of 2.5 times or more were shown. **b** David enrichment analysis showed that cytokines and cytokine receptors were highly correlated with 4T1 lung metastasis.


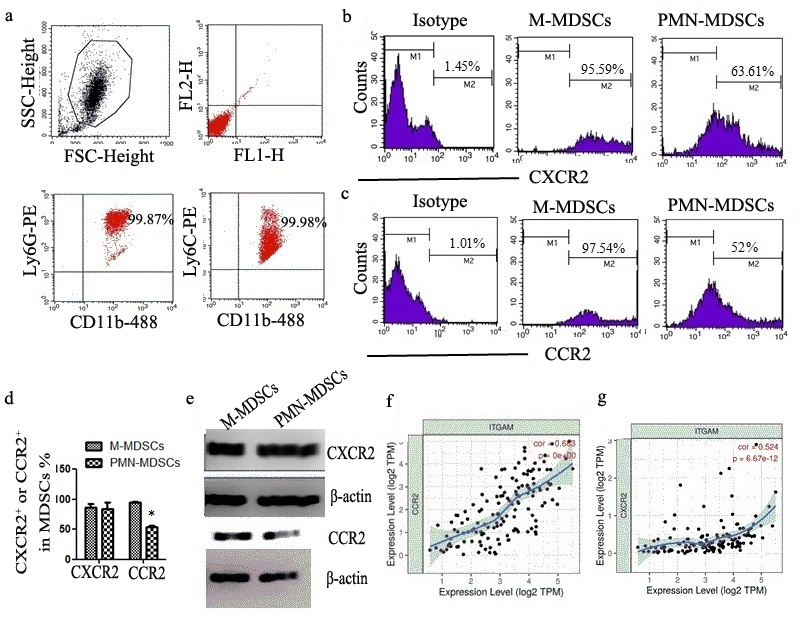


**SFig. 2** CXCR2 and CCR2 expression in MDSCs. **a** Purity analysis of MDSCs derived from 4T1 tumor-bearing mice. Mononuclear cells from lung tissue of 4T1 tumor-bearing mice were obtained by Ficoll density gradient centrifugation. Then CD11b^+^ cells were obtained from lung mononuclear cells by sorting flow cytometry. Finally, Ly6G^+^ PMN-MDSCs or Ly6C^+^ M-MDSCs were sorted by immunomagnetic beads in CD11b^+^ cells. Purity was analyzed by flow cytometry. **b,c,d** and **e** CXCR2 or CCR2 expression on M-MDSCs/PMN-MDSCs in mononuclear cell of 4T1 tumor-bearing mice lung tissues. **f** and **g** Correlation of CCR2 and CXCR2 with CD11b^+^ myeloid cells infiltration in basal breast cancer was analyzed via the Oncomine database and Tumor Immune Estimation Resource (TIMER) site. CXCR2 and CCR2 showed significant positive correlation with CD11b^+^ myeloid cells.


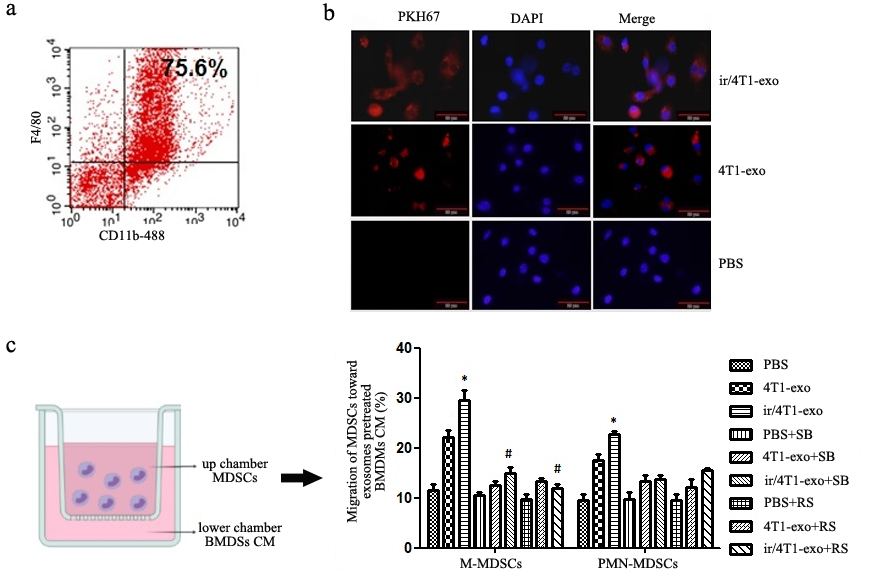


**SFig.3** Conditioned medium (CM) of ir/4T1-exo pretreated BMDMs promotes the migration of M-MDSCs. **a** Bone marrow cells of BALB/c mice were treated with M-CSF for 9d to induce bone morrow derived macrophage (BMDMs) differentiation, then, CD11b^+^F4/80^+^ BMDMs frequency was detected by flow cytometry. **b** Exosomes were isolated from 4T1 cells (4T1-exo) or 20Gy radiation irradiated 4T1 cells (ir/4T1-exo) using the ultracentrifugation method and labeled with PKH26. Uptake of PKH26-labeled exosomes at 37 °C for 7h and observed by fluorescence microscope. **c** BMDMs were pretreated with additional 4T1-exo or ir/4T1-exo (50μg/ml) at 37 °C for 24 h, followed by wash and 48 h culture for CM collection. The migration of M-MDSCs and PMN-MDSCs were measured by transwell. Compared with PBS pretreated CM, * *p*<0.05; Compared with ir/4T1-exo pretreated CM, # *p*<0.05.
